# Remote Examination and Screening for Domestic Abuse. Comment on “Online Antenatal Care During the COVID-19 Pandemic: Opportunities and Challenges”

**DOI:** 10.2196/23295

**Published:** 2021-02-17

**Authors:** Hannah Lee Grimes, Ramnik Uppal

**Affiliations:** 1 University of Cambridge Clinical School Cambridge United Kingdom

**Keywords:** spouse abuse, domestic abuse, apps, patient information, antenatal care, COVID-19

After reading the viewpoint by Wu et al [1] on opportunities for the delivery of antenatal care online during the COVID-19 pandemic, we would like to draw attention to a potential further aspect of this analysis. The authors surveyed 983 Chinese pregnant women and concluded that online antenatal care is likely to be an effective alternative for pregnant women, enabling them to access basic care without attending the hospital[1]. This is optimal with regards to minimizing the infection risk during the pandemic. However, it is important to remember that for a proportion of women, these antenatal visits provide a critical opportunity for physicians to screen for domestic abuse. 

A cross-sectional study, also assessing a population of Chinese pregnant women, found that 15.62% of their subjects were identified as victims of domestic abuse [2]. Data released by the World Health Organization suggest that the number of women affected by domestic violence during pregnancy may be between 1% and 28% [3]. This proportion is by no means negligible. 

In many countries, antenatal screening for domestic abuse when consulting with the patient is sporadic. One study of Canadian doctors found that 33% of professionals surveyed “never or only rarely” asked women about domestic abuse during antenatal appointments [4]. Provision of antenatal care online could result in even fewer women being able to confide in their doctor and receive much-needed assistance with respect to abusive situations at home. This might be due to women feeling less able to seek help through an online service compared to face-to-face consultations.

The COVID-19 pandemic has complicated this situation further. The London metropolitan police reported that since lockdowns were introduced in the United Kingdom, 380 more calls per week related to domestic abuse have been recorded [5]. Unpacking this data further, it is noteworthy that the majority of these reports of abuse were from third-party callers, perhaps due to the increased presence of neighbors [5]. This suggests that while there may be a potential surge in domestic abuse due to lockdown restrictions, this may not be associated with an increased probability of victims reporting the crime.

We suggest that to best address this gap in their proposal, Wu et al [1] should factor in how their proposed online antenatal care delivery system could incorporate adaptations to provide help to victims without compromising their safety. One possible method of achieving this could be to add a layer to any application developed through which women could find support numbers for relevant domestic abuse charities or alert their doctor to their need for a face-to-face appointment to discuss their home situation. It is important for this feature to be sufficiently blended into the app such that it would not alert suspicion from the perpetrator and thus compromise the safety of any woman seeking help.
